# Supplementary material for: Beliefs and socio-cultural perspectives on hantavirus in a rural community in Panama: An ethnonursing study
Source: PLOS Glob Public Health. 2025 Oct 22;5(10):e0005320. doi: 10.1371/journal.pgph.0005320 (PMC12543134; doi:10.1371/journal.pgph.0005320)
Supplement: S1 File — (DOCX) [file pgph.0005320.s001.docx]

***GENERAL INTERVIEW GUIDE FOR KEY AND GENERAL PARTICIPANTS***

Interviews were conducted when evidence of the participant’s transition from stranger to trusted friend was obtained, according to Leininger (2006). Each interview was carried out in the following three phases:

**Phase 1: Introduction to the interview**

In this first phase, the interviewer explained the dynamics of the interview to the participant. The interview began with a warm greeting, such as “How are you today?” or “How have you been in the last few days?”. The interviewer then described the purpose of the interview, its approximate duration, the fact that it would be recorded, and the fact that the recorded audio would be deleted after the interview was transcribed. Finally, the interviewer explained the contents of the informed consent form and gave each participant time to read and sign it. During this phase, fluid, friendly, and confident communication was encouraged.

**Phase 2: Development of the interview**

In this second phase, the interviewer asked the participant open-ended questions, considering the phenomenon under study, Leininger’s sunrise model, and the research question. The nature of the questions asked was open, leaving room for the formulation of new questions based on the participant’s responses. Sociodemographic questions to characterize the participants were also formulated in this phase.

**Phase 3: Closing and farewell**

In this third phase, the interviewer ended the interview and thanked the participant for participating in the study. The participant was asked if he or she had any questions about the study and if the interviewer (a nurse) could help with anything.

***QUESTIONS ABOUT THE PARTICIPANTS’ CHARACTERISTICS***

1. **Key participants:** age, gender, education, years living in the community, personal or family history of hantavirus.
2. **General participants:** age, gender, role or activity within the community.

***QUESTIONS ABOUT THE STUDY TOPICS***

1. How long have you lived in the Bebedero Tonosí community? What is it like to live in this community? Can you tell me about the life, work, and people in the community?
2. Can you tell me about your family and friends in the community? How do you communicate with each other?
3. Can you tell me about when you first heard about hantavirus? What have been your experiences with hantavirus? What about your family, friends, and neighbours?
4. How do you think hantavirus is transmitted? What do you do to prevent it?
5. If you or someone in your family has become ill with hantavirus, how did it happen and what did you and your family do about it?
6. How has your family and community taken care of or prevented hantavirus illness (in the past and now)? What happens if someone in your family or a friend gets hantavirus?
7. What do you think it can be done to prevent hantavirus?
