# Supplementary material for: Beliefs and socio-cultural perspectives on hantavirus in a rural community in Panama: An ethnonursing study
Source: PLOS Glob Public Health. 2025 Oct 22;5(10):e0005320. doi: 10.1371/journal.pgph.0005320 (PMC12543134; doi:10.1371/journal.pgph.0005320)
Supplement: S4 File — (DOCX) [file pgph.0005320.s004.docx]

***KEY AND GENERAL PARTICIPANTS CHARACTERISTICS***

*Key participant characteristics*

| **ID** | **Age (range)** | **Gender** | **Education** | **Personal or family history of hantavirus** | **Length of residence** |
| --- | --- | --- | --- | --- | --- |
| K01 | < 60 | Male | No education | Family | Entire life |
| K02 | 41-50 | Female | University | Personal | Entire life |
| K03 | 51-60 | Female | Primary | Family | 22 years |
| K04 | 51-60 | Female | Primary | Family | Entire life |
| K05 | 41-50 | Female | University | Family | 15 years |
| K06 | 41-50 | Female | University | Family | 15 years |
| K07 | 30-40 | Female | Secondary | Family | 20 years |
| K08 | 51-60 | Female | Secondary | Personal | 10 years |
| K09 | 41-50 | Female | Secondary | Family | 12 years |
| K10 | < 60 | Female | No education | Personal | Entire life |
| K11 | 51-60 | Female | University | Family | Entire life |

Note. K = Key participant.

*General participant characteristics*

| **ID** | **Age (range)** | **Gender** | **Role in the community** |
| --- | --- | --- | --- |
| G01 | 30-40 | Female | Health system |
| G02 | 41-50 | Female | Health system |
| G03 | 30-40 | Female | Health system |
| G04 | 30-40 | Male | Health system |
| G05 | 30-40 | Female | Health system |
| G06 | 51-60 | Female | Health system |
| G07 | 30-40 | Male | Health system |
| G08 | 30-40 | Male | Health system |
| G09 | 41-50 | Female | Health system |
| G10 | > 30 | Female | Health system |
| G11 | 30-40 | Male | Health system |
| G12 | 30-40 | Female | Health system |
| G13 | 41-50 | Male | Community organisation |
| G14 | 30-40 | Female | Community organisation |
| G15 | < 60 | Female | Community organisation |
| G16 | 41-50 | Male | Community organisation |
| G17 | <60 | Male | Community organisation |
| G18 | 41-50 | Female | Health system |
| G19 | < 60 | Male | Health system |

Note. G = General participant.
