# Supplementary material for: Beliefs and socio-cultural perspectives on hantavirus in a rural community in Panama: An ethnonursing study
Source: PLOS Glob Public Health. 2025 Oct 22;5(10):e0005320. doi: 10.1371/journal.pgph.0005320 (PMC12543134; doi:10.1371/journal.pgph.0005320)
Supplement: Attachment — Submitted filename: Response to Reviewers.docx [file pgph.0005320.s006.docx]

Najmul Haider, PhD
Academic Editor
PLOS Global Public Health

Dear Dr. Haider

We would like to thank you and the reviewers for your feedback on our manuscript entitled “*Beliefs and socio-cultural perspectives on hantavirus in a rural community in Panama: an ethnonursing study*” and for the opportunity to submit a revised version of the manuscript. As requested, we have revised the manuscript based on your suggestions, and we believe that these revisions have improved the overall quality of the manuscript. To facilitate the review process, we have prepared a table describing our revisions, with our responses to each reviewer and each comment. We have also highlighted in yellow the changes we have made to the manuscript.

| **Reviewer Comments and Author Responses** | | |
| --- | --- | --- |
| **Editor** | | |
| 1 | Thank you for submitting your manuscript to PLOS Global Public Health. After careful consideration, we feel that it has merit but does not fully meet PLOS Global Public Health’s publication criteria as it currently stands. Therefore, we invite you to submit a revised version of the manuscript that addresses the points raised during the review process. | We appreciate your positive comments regarding our article. We believe this study contributes to public health by emphasizing the significance of beliefs and sociocultural perspectives in preventing hantavirus. We hope this study will strengthen prevention strategies for hantavirus and guide future studies on other zoonoses. |
| **Reviewer 1** | | |
| 2 | This is an excellent and important study. With some revisions for clarity, consistency, and elaboration in key areas, the manuscript will make a valuable contribution to the literature on hantavirus and community-based health perspectives. | Thank you for your kind comments. |
| 3 | **Abstract**  The host of hantavirus could be briefly mentioned to better align the abstract with the socio-ecological drivers that facilitate spillover. | We clarified the host of the hantavirus by explaining that “the disease is transmitted to humans through the inhalation of aerosols containing feces, urine, and saliva from asymptomatic infected rodents” (Page 2). |
| 4 | The study timeline is missing and should be included. | We indicated that the study timeline was from September 3, 2021, to August 25, 2022 (Page 2). |
| 5 | More detail is needed on participant selection: Who were the participants, how were they selected, and why? It is not immediately clear who were considered general participants versus key participants | We indicated the inclusion criteria for key and general participants. Key participants were community members with personal or familial experience with hantavirus. General participants included community authorities and health professionals. Due to the lack of space in the abstract (currently 290 out of 300 words), we have not provided further details (Page 2). |
| 6 | The selection criteria for key participants should be explained participation in the prior quantitative study alone does not sufficiently justify their inclusion | As previously mentioned, we explained that the key participants had personal and/or familial experiences with hantavirus. These experiences qualify them as key informants for the present study, which aims to explore beliefs and care practices related to hantavirus (Page 2). |
| 7 | A brief recommendation should be added to the abstract for completeness | We indicated that the study findings were used to propose a model for decision and action in hantavirus prevention. This model emphasises the importance of implementing prevention practices that are relevant, sustainable, and culturally aligned with the affected communities (Page 2). |
| 8 | **Introduction**  There is inconsistency in terminology: the abstract refers to "Hantavirus Pulmonary Syndrome" while the introduction uses "Hantavirus Cardiopulmonary Syndrome". Please ensure consistent terminology throughout | We have made the terminology consistent by always using the term “Hantavirus Pulmonary Syndrome”, which is more comprehensive and widely used internationally. Initially, we used the term “Hantavirus Cardiopulmonary Syndrome” because it is commonly used in South American countries and Panama, where cardiac and respiratory involvement is more prevalent in reported cases (Page 4). |
| 9 | While the authors mention the socio-cultural dimensions of hantavirus, additional justification is needed in the introduction to explain how this study adds to the existing literature | We explained that this study adds to the existing literature on hantavirus by providing a comprehensive understanding of the disease, incorporating the perspectives of those living in the affected communities, including their care practices, the beliefs associated with these practices, and the broader sociocultural context. We emphasized that this approach may enable the design and implementation of effective, context- and culture-specific prevention programs (Page 6). |
| 10 | Leininger’s Theory of Culture Care Diversity and Universality is mentioned multiple times and could be referenced more concisely. | We explained that Leininger’s Theory of Culture Care Diversity and Universality draws from both anthropology and nursing. We made it clear that anthropology provides an understanding of health and illness from a sociocultural perspective, while nursing uses this understanding to deliver culturally adapted care (Page 5). |
| 11 | The ethnonursing approach should be elaborated further to clarify its application. | In the Methods section, we clarified that the method was created by the nurse and anthropologist Madeleine Leininger, and that it takes a naturalistic, open and inductive approach to exploring beliefs, experiences and cultural practices related to health and illness (Page 7). |
| 12 | **Methods** The study timeline (September 3, 2021, to August 25, 2022) should be moved to the beginning of the Methods section. | We moved the study timeline to the beginning of the Methods section (Page 7). |
| 13 | The use of Leininger’s Stranger to Trusted Friend Enabler needs more elaboration—how and why was it applied? | We provided more details on this tool by explaining that it contains a set of qualitative, observable indicators that allow researchers to assess participants’ trust in them. We made it clear that this trust is expressed through observable behaviors, emotional expressions, and a willingness to communicate. Once trust is established, researchers transition from being strangers to trusted friends, thereby facilitating the collection of more accurate and meaningful data (Page 8). |
| 14 | Provide more clarity on key participant selection criteria, including why they were selected and how their involvement contributes uniquely to the study | In the Participants section, we outlined the selection criteria for key and general participants. We explained why it is important to consider the views of both the affected population and health professionals and community authorities in order to gain a more comprehensive understanding of the disease (Pages 8 and 9). |
| 15 | A good description is provided for general participants. | Thank you for your positive comment. |
| 16 | The process of theoretical saturation should be explained. Was data analysed during fieldwork, and what strategies were used to determine when saturation was reached? | We explained that, in line with the iterative nature of the qualitative design, interviews and data analysis were conducted concurrently, with each interview analyzed immediately afterwards. As the analysis progressed, the findings informed the selection of new participants who could provide further information on particular patterns, while adhering to the previously described participant selection criteria. When information was repeatedly expressed by multiple participants and no new patterns were identified in the data analysis, it was deemed that saturation had been reached and recruitment of new participants was stopped (Pages 12 and 13). |
| 17 | **Results** Under participant characteristics, it may not be necessary to separate key participants and general participants in the analysis—they could be presented together unless a clear justification is provided for treating them separately. | Thank you for pointing that out. The distinct characteristics of each participant group warrant presenting the results separately for each. Key participants are residents of areas where hantavirus occurs, with personal or familial experiences with the disease. In contrast, general participants include health professionals experienced in treating and preventing the disease, as well as community authorities with knowledge of the disease and the social dynamics within the community. In addition, reporting the findings separately for each group aligns with the approach taken in previous ethnonursing studies. In addition, we added a supplementary file with the individual characteristics of each study participant (Page 14 and SI3). |
| 18 | **Discussion** The discussion is well-structured; however, in several places, the results are repeated without deeper interpretation. Consider focusing on interpreting the findings rather than reiterating them. | Thank you for this comment. We have made several changes throughout the discussion with the aim of focusing on the interpretation of the findings. |
| 19 | The authors should explain why participants hold certain beliefs and discuss their implications for risk perception, prevention, and control | In line with previous studies, we explained that beliefs that are inconsistent with scientific literature could hinder the adoption of vector control, the primary preventive measure against hantavirus (Pages 26 and 27). |
| 20 | The study site name, currently mentioned in the discussion, should be included earlier in the Methods section | In the Methods section, we made it clear that the study was conducted in a community in the district of Bebedero, in the province of Los Santos, Panama (Page 7). |
| 21 | Please address the potential bias introduced by selecting participants from the prior quantitative study. Their prior exposure to structured questions and potential discussions with peers may have influenced their knowledge or perception of hantavirus. | We noted that key participants in the qualitative phase may have introduced bias into their responses because they had already been exposed to the topic in the quantitative phase. However, we clarified that interviewing participants who have previously participated in a quantitative phase is a legitimate strategy in explanatory sequential mixed methods designs because it allows the quantitative findings to be explored in more depth. Additionally, we emphasized that the selected participants had close personal and familial experience with hantavirus. Therefore, regardless of their prior participation in the survey, these individuals had solid knowledge of and experience with the topic, making it highly likely that their responses would have been similar had they not completed the survey (Page 35). |
| 22 | Recommendations should be more specific, particularly regarding what is needed to address gaps in the existing health infrastructure. | From a sociocultural perspective, we emphasized the importance of implementing the model for decision and action in hantavirus prevention proposed in this study to promote active community participation in designing and implementing hantavirus prevention programs. This approach ensures that these programs are relevant, sustainable, and culturally appropriate (Pages 31-34 and 36). |
| **Reviewer 2** | | |
| 23 | The article provides a very well-written depiction of community’s beliefs about the modes of hantavirus transmission and the care and prevention practices adopted in accordance with these beliefs. Most importantly, the authors leveraged the findings to provide important insights for the development of hantavirus prevention education programmes aligning with the social and cultural beliefs and norms, and the socio-economic context of community members | We appreciate your positive feedback on the paper. We believe that people's beliefs, lifestyles and sociocultural context influence the preventive practices they adopt to protect themselves from disease. We also believe that this knowledge is vital for creating interventions that are socially and culturally acceptable. We are pleased to have conveyed this idea accurately in our article. |
| 24 | I am not confident about how well-balanced the amount of details in theme 2, particularly pattern 2.2, is in respect to its interpretation in the discussion and direct link with the understanding of issues related to hantavirus disease. The fifth paragraph of the discussion section might be revisited to more explicitly interpret the linkage between hantavirus infection and community’s practice and perception related to agricultural and rural work in light of findings in other contexts. | In the Discussion section, we expand on the profound cultural roots of agriculture and consider how health prevention measures might affect long-standing agricultural traditions. We emphasize that respectful dialogue between community members and health professionals is essential for effectively implementing and accepting these measures (Pages 28-29). |
| 25 | Gender perspective seems to be missing. Given the gender imbalance in key participants (mostly women), I am afraid that there would be important perspective missed from men, who are often important role players or decision makers in many rural communities. It should at least be addressed in the limitations. | In the Participants section, we explained that the criterion of participant diversity in terms of gender (male/female) was not met, as most of the men contacted refused to participate due to lack of time. In the Limitations section, we mentioned the low male participation among key participants and note that it may affect the depth of the analysis from a gender perspective (Pages 8, 9, and 35). |
| 26 | Methods section could include information on how participants were recruited based on age or gender. | We explained that diversity among key participants was sought intentionally. During the recruitment process, we contacted individuals who met the inclusion criteria. We made it clear that, except for gender, we achieved diversity in all areas, given the low participation of men due to time constraints (Pages 8 and 9). |
| 27 | The discussion and conclusion sections could be further improved by minimizing repetition of results and incorporating more critical discussion in light of situation in other countries/context | As we mentioned in our response to Reviewer 1, we placed a greater emphasis on interpreting the findings in the Discussion section, particularly in the summary of findings. We also emphasized the importance of implementing the model for decision and action in hantavirus prevention in order to promote active community participation in designing and implementing hantavirus prevention programs (Pages 31-34 and 36). Lastly, we discussed how the findings of this study relate to those of other studies on hantavirus and other zoonoses conducted in different geographical contexts (Page 31). |
| 28 | Instead of including description of individual participants, table 1 and 2 could be combined to present summarized description | Thank you for pointing this out. To improve the reporting of participants’ characteristics, we merged Tables 1 and 2 into a single summary table. We maintained the distinction between key and general participants because their selection criteria were different. We added a supplementary file with the individual characteristics of each study participant (Page 14 and SI3). |
